# Proteomic insights into SARS-CoV-2 infection mechanisms, diagnosis, therapies and prognostic monitoring methods

**DOI:** 10.3389/fimmu.2022.923387

**Published:** 2022-09-20

**Authors:** Shengman Yu, Xiaoyan Li, Zhuoyuan Xin, Liyuan Sun, Jingwei Shi

**Affiliations:** ^1^Department of Laboratory Medicine Center, China-Japan Union Hospital, Jilin University, Changchun, China; ^2^School of Laboratory Medicine, Beihua University, Jilin, China; ^3^Department of Infection Control Department, Hospital of Stomatology, Jilin University, Changchun, China; ^4^The Key Laboratory of Zoonosis Research, Chinese Ministry of Education, College of Basic Medical Science, Jilin University, Changchun, China

**Keywords:** proteomics, COVID-19, SARS-CoV-2, protein biomarker, virus infection

## Abstract

At the end of 2019, the COVID-19 pandemic, caused by severe acute respiratory syndrome coronavirus type 2 (SARS-CoV-2) infection, seriously damaged world public health security. Several protein markers associated with virus infection have been extensively explored to combat the ever-increasing challenge posed by SARS-CoV-2. The proteomics of COVID-19 deepened our understanding of viral particles and their mechanisms of host invasion, providing us with information on protein changes in host tissues, cells and body fluids following infection in COVID-19 patients. In this review, we summarize the proteomic studies of SARS-CoV-2 infection and review the current understanding of COVID-19 in terms of the quantitative and qualitative proteomics of viral particles and host entry factors from the perspective of protein pathological changes in the organism following host infection.

## Introduction

Severe acute respiratory syndrome coronavirus 2 (SARS-CoV-2) was identified as a new type of β coronavirus in 2019. Infection with SARS-CoV-2 in humans has rapidly spread around the globe (1, 2). The WHO named the resulting disease coronavirus disease-19 (COVID-19) in March 2020 (3, 4), and later declared the COVID-19 outbreak to have reached pandemic status, which attracted worldwide attention. At the genomic level, SARS-CoV-2 is highly similar to SARS-CoV, but it is far more transmissible (1). The virus spreads mainly *via* respiratory droplets and invades human bronchial and lung epithelial cells. SARS-CoV-2 usually causes lower respiratory tract infections (4, 5), which can progress to severe acute respiratory syndrome and even multiple organ failure (6, 7). Since reaching epidemic status, SARS-CoV-2 has continuously mutated from the initially discovered wild-type strain to produce alpha, beta, gamma, delta, and the recently emerged OMICRON variants due to the instability of RNA viruses (8). Epidemiology data reported to the WHO (https://covid19.who.int/) showed 590,659,276 confirmed cases of COVID-19 globally as of 5:15 pm CET on 18 August 2022, including 6,440,163 deaths, and these cases have seriously impacted public health security worldwide. Thus, effective preventive, control, and intervention measures are urgently needed. Exploring the pathogenic mechanism of the virus and the physiological and pathological state of the human body after infection by proteomic methods will help deepen our understanding of COVID-19 and will be beneficial for subsequent treatments and vaccine development (9–11).

## Overview of COVID-19 proteomics

The infection mechanism and clinical symptoms of COVID-19 are complex, and scientists around the world have been devoted to exploring the scientific answers behind these complex clinical questions since the epidemic began. The symptoms of SARS-CoV-2 infection range from single respiratory symptoms to dysfunction involving multiple organs throughout the body, and asymptomatic infection poses challenges to research and clinical practice (12–15). At present, a variety of omics methods have been used to explore COVID-19, and genome, transcriptome, proteome, and metabolome analyses have jointly improved our understanding of this virus and its associated diseases, providing us with a multidimensional perspective (16–19). In particular, proteomics uses proteomic approaches to focus on annotation, posttranslational modifications, and the interactions of proteins encoded by genomic sequences (20). Mass spectrometry is the most widely used proteomics technology in proteomics research, especially matrix-assisted laser desorption/ionization mass spectrometry (MALDI-TOF MS) (21, 22) and electrospray ionization mass spectrometry (ESI-MS) (23, 24), which are biological mass spectrometry technologies and play an extremely important role in the detection of SARS-CoV-2 infection-related markers.

## Qualitative and quantitative proteomics

Basic qualitative proteomics and quantitative proteomics studies provided us with the relevant polypeptide levels of SARS-CoV-2 and its host entry factors, as well as the distribution and changes of host entry factors. These data allow us to characterize viral and host entry factor peptides. In an earlier study, tryptic viral peptides and tryptic host cell peptides were characterized to recommend a list of peptides that can be used for the targeted detection and parallel reaction monitoring of viral proteins (23, 25). Peptide detection of ACE2 and TMPRSS2, the main host entry factors of SARS-CoV-2, has also been attempted (25, 26), but the outcome was not satisfactory. This failure may be attributed to the different expression profiles of ACE2 and TMPRSS2 in various tissues and cell lines (25–28), and most current studies suggest that the severity of SARS-CoV-2 infection differs from receptor expression (29). Collectively, existing studies have shown that ACE2 and TMPRSS2 are difficult to detect in upper respiratory tract and lung tissue samples (27, 28, 30). For example, in the human lung, adenocarcinoma H522 cells do not express ACE2 (31),but ACE2, a SARS-CoV-2 receptor, is often affected by different factors during infection, such as the dysregulation of interferon signaling pathways (32, 33). Another typical example is intestinal tissue. The Caco-2 cell line is often used to study the mechanisms and symptoms of SARS-CoV-2 infection, and ACE2 and TMPRSS2 can be codetected in multiple intestinal samples (27, 28, 30).

A major goal of qualitative proteomics is to find markers with clinical diagnostic potential, while quantitative proteomics can quickly provide us with useful information because of its simple operation and easy interpretation of results (34). The general quantitative methods for protein biomarker identification are usually divided into targeted and nontargeted methods. Targeted quantification is a biased strategy that only focuses on the identification of a small subset of candidate biomarkers. Targeted quantification is available to provide a complete secondary mass spectral profile for confirming the identity of the target and quantitative information. For example, parallel reaction monitoring (PRM) can selectively quantify target proteins and peptides in absolute or relative terms with a high level of accuracy, reproducibility and sensitivity (35, 36). Nontargeted quantification can be divided into two categories: labeled and unlabeled. Widely used tags are isobaric tags for relative and absolute quantification (iTRAQ) and tandem mass tags (TMTs). TMTs are often combined with high-throughput mass spectrometry platforms for the analysis of a wide range of clinical samples, such as liquid chromatography-tandem mass spectrometry (LC−MS/MS) (37, 38). Simultaneous marker quantification is now possible for up to 16 markers, making it particularly suitable for differential proteomic analysis of COVID-19 samples using multiple treatments or from multiple treatment times. Data-independent acquisition (DIA) is a commonly used label-free quantification method, such as the sequential window acquisition of all theoretical fragment ions (SWATH) for large-scale label-free quantification, which can efficiently determine protein molecules of very low abundance in complex samples with good reproducibility (39). The advent of another revolutionary new method, parallel accumulation serial fragmentation (PASEF), has endowed shotgun proteomics with greater speed and sensitivity, making it more suitable for the analysis of microsamples (40). In addition, patented proximity extension assay (PEA)-based technology empowers Olink proteomics solutions to combine the high specificity of antibody immunoassays with the high sensitivity and throughput of genomics (41). Furthermore, this method is particularly suitable for the analysis of plasma serum samples. These multiple proteomics strategies and technologies all provide powerful tools for us to explore the SAS-CoV-2 infection pathway, identify therapeutic targets and screen drugs.

## Host cell proteomics

### Cell- and tissue-based studies

**Figure 1:**
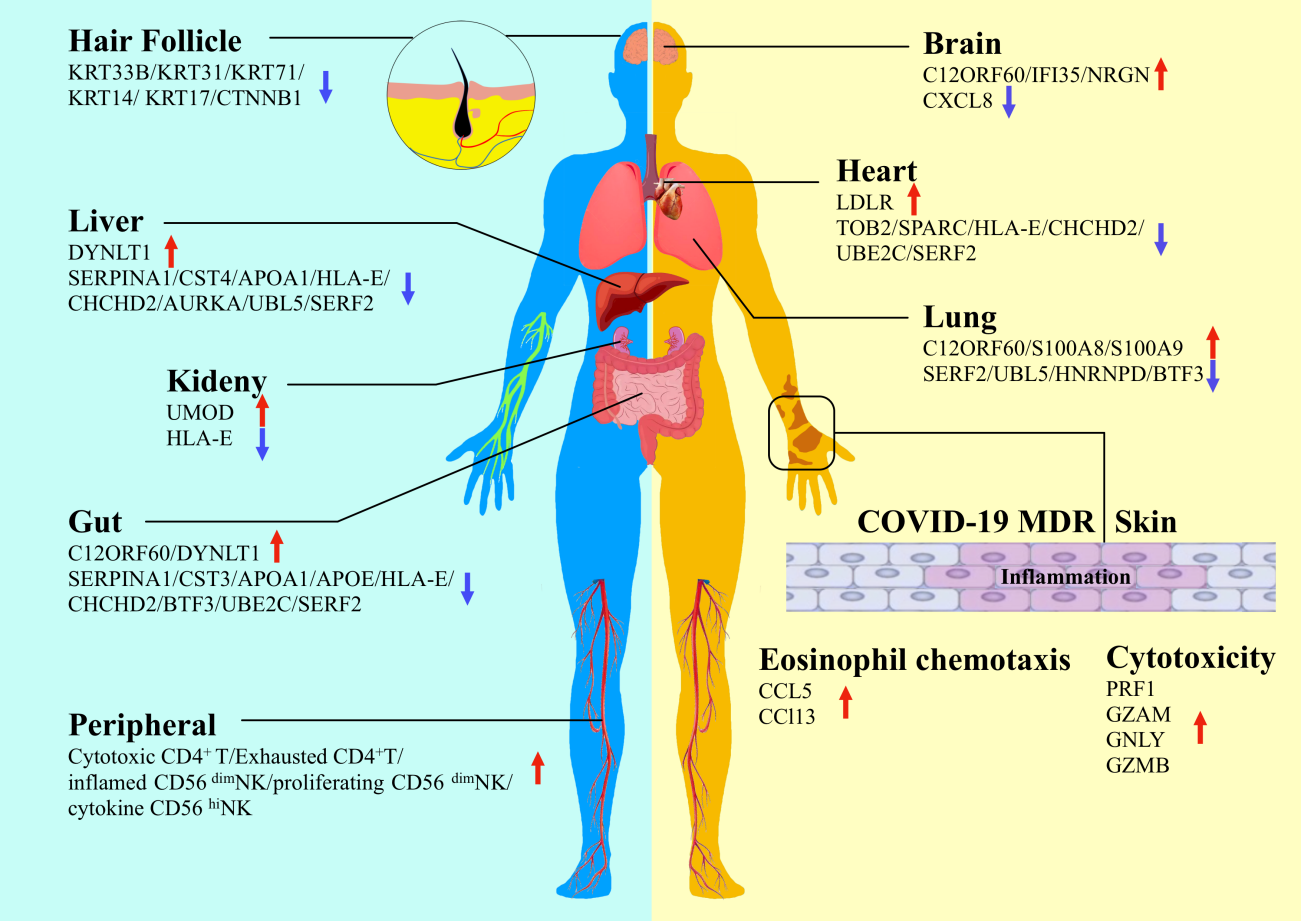
Proteomics study detects variations of protein expression in different organs infected by SARS-CoV-2 (Hair follicle, brain, heart, lung, liver, kidney, gut, skin and peripheral are shown in schematic).

Although SARS-CoV-2 invades the respiratory tract, with the lungs and bronchi being the most important sites of infection, a growing number of studies of tissue and autopsy collected from infected individuals have shown that SARS-CoV-2 infection is present in multiple organs due to viral replication, including the gut, liver, kidney, myocardium, brain and nerves (4, 42–46). Numerous studies have shown variations in protein expression in different organs infected with SARS-CoV-2 (**Figure 1**). By conducting a comprehensive investigation of the pathways by which viruses destroy cells, Chen et al. (47) established a unique cell culture model of six human cell lines to analyze the cellular response to of infection in the lung, liver, intestine, kidney, heart, and brain based on proteomics. They found that SARS-CoV-2 targeted proximal jak-stat pathway components and destabilized type-I interferon receptors through ubiquitination, thereby making infected cells resistant to type-I interferons, revealing the immune evasion strategy of SARS-CoV-2. By analyzing the SARS-CoV-2-infected cell landscape, Pablos et al. (48) identified more than 100 substrates in human lung and kidney cells and delineated the human protein substrates of 3clpro by tail substrate-targeting N-terminal omics. They found that 3clpro targeted the Hippo pathway, including MAP4K5 inactivation and the key effectors of mRNA transcription, mRNA processing, and mRNA translation. They demonstrated that the spike glycoprotein directly bound galectin-8, and galectin-8 cleaved CALCOCO2/NDP52 to decouple antiviral autophagy.

The symptoms of SARS-CoV-2 infection are not limited to the respiratory tract. Common symptoms include fever, cough, fatigue, dyspnoea, and decreased taste/smell. Multiple organ damage is a major complication of COVID-19. Qiu et al. (49) used tandem mass tag (TMT) 11-plex labeling and liquid chromatography with tandem mass spectrometry (LC−MS/MS) to analyze different tissues and organs of deceased patients. They identified potential tissue-specific proteins and different molecular markers for different tissue types. The most typical of these markers is S100A8/9, which is significantly elevated in the lung tissues of patients with severe COVID-19. In addition, dermatological manifestations are widely reported among the extrapulmonary signs associated with COVID-19 (50). In an interesting study, the impact of COVID-19 on the development of macular drug eruption (MDR) was explored in an attempt to address the difference between COVID-19 patients suffering from MDR (COVID-MDR) and non-COVID-19 patients suffering from MDR or patients with drug rash with eosinophilia and systemic symptoms (DRESS) (51). Proteomic analysis showed that a large-scale systemic cytokine storm emerged in COVID-MDR, which was distinct from MDR and DRESS. However, SARS-CoV-2 RNA was not detected in the skin of patients with COVID-MDR, and ACE2 was regulated at the mRNA rather than the protein level (51). One of the significant findings is that the COVID-MDR cases were all severe COVID-19, and all patients developed MDR approximately one month after the initial COVID-19 diagnosis. The results of this study suggest that MDR in critically ill COVID-19 patients may be attributed to a hyperinflammatory immune response that ultimately leads to the activation of Mos/Macs and highly cytotoxic CD8+ T cells. Further studies in larger patient populations are needed in the future. In a recent study of SARS-CoV-2 infection in skin tissues and organs, Ma et al. (52) investigated the pathological mechanism of SARS-CoV-2 infection based on a 3D skin model for the first time. The authors constructed a human-induced pluripotent stem cell (hiPSC)-derived skin organoid model with hair follicles and a nervous system and developed a skin proteomics atlas of SARS-CoV-2 patients by quantification methods. They found that many organs and tissues were affected by COVID-19 infection, including the extracellular matrix, epithelial development, the nervous and circulatory systems, and the cellular connections of the skin. Their results show that SARS-CoV-2 can directly attack hair follicles, which provides evidence for an association between COVID-19 and the sequelae of hair loss. They also found that SARS-CoV-2 can attack neurons in the skin. These findings demonstrate the great potential and value of skin organoids in pathological mechanism research and drug screening.

Severe SARS-CoV-2 infection triggers a series of pathophysiological processes in the body. Thus, elucidating the pathogenic mechanisms of infection and controlling host immunity during infection are vital to resist viral infection without overreaction. Innate and adaptive immunity interact and counteract each other and defend against viral invasion. A recent study showed that extensive T-cell apoptosis may be a key factor in T-cell homeostasis and dysfunction in severe COVID-19 patients. Adamo et al. (53) analyzed T-cell phenotype and function in severe COVID-19 patients using flow cytometry and targeted proteomics and found a significant loss of peripheral T lymphocytes in naive and memory populations in patients suffering from severe COVID-19 infection, particularly an age-related loss in CD8+ T cells, accompanied by an increase in interleukin-7 and increased T-cell proliferation, which are consistent with previous findings (54–56). These findings revealed the importance of COVID-19-induced disorders of CD4+ and CD8+ T cells and demonstrated the association of age and T lymphocytes with COVID-19 severity. Therefore, targeting T lymphocytes and inflammation has great potential for therapeutic value. Two studies have focused on NK-cell immunity (57, 58). By analyzing the relationship between NK-cell-related immune signatures and viral load using a single-cell count-based proteomics platform, Hsieh et al. (57) showed that the diversity of NK receptors was negatively correlated with viral clearance and that patients with a higher abundance of the NK subset expressing DNAM1 and its paired coinhibitory receptor TIGIT had faster viral clearance than patients with lower levels of these receptors. Furthermore, CD155 and nectin-4 preferentially bound to the ligands of DNAM 1 paired receptors, which are upregulated after SARS-CoV-2 infection and can regulate the interaction between NK cells and virus-infected cells. Krämer et al. (58) further explored the impact of SARS-CoV-2 infection on the function and composition of NK cells and found that differences in ifn-α and TNF responses were an important mechanism in the early stages of COVID-19. The authors described the link between excessively prolonged ifn-α-induced NK-cell responses and persistent NK cells with cellular dysfunction of adverse courses, such as pulmonary fibrosis in severe COVID-19.

### Body fluid-based studies

Plasma-based proteomics can be a rapid, noninvasive method for understanding the SARS-CoV-2 infection process and its physiological effects on the host (59). Plasma and circulating markers have previously been used to study protein differences between healthy individuals and COVID-19 patients with varying severity and to serve as the predictors of COVID-19 surveillance (37, 38, 60). Several new research advances are emerging to focus on this aspect (**Table 1**). Alaiya et al. (66) provided a method to objectively predict disease outcome in severe COVID-19 patients using LC−MS/MS for identifying and quantifying proteomic expression in different degrees of COVID-19 peripheral blood samples from patients. They identified 49 differentially expressed proteins that were upregulated in the peripheral blood of critically ill patients; 9 of them were upregulated in asymptomatic patients, and 12 of them had predictive value for recovery from severe disease. Further analysis revealed that these proteins are associated with several pathways, such as humoral limmunity, inflammation, acute-phase response signaling, liver X receptor/retinol X receptor (LXR/RXR) activation, coagulation, and the complement system. In another study, Villar et al. (61) employed sequential windowed data independent acquisition of the total high-resolution mass spectra (SWATH-MS) quantitative serum proteomics and multiple data analysis algorithms to investigate the host response to SARS-CoV-2 infection in different cohorts ranging from asymptomatic individuals to critically ill patients. They identified immune-related prognostic biomarkers and physiological processes associated with the symptoms of COVID-19 infection. Their results showed that the disease recovery biomarkers SELENOP and PON1, the severity marker CBP2, and the symptomatic marker PZP can be used for disease prognosis. Sarif et al. (62) proposed that soluble uPAR could be a useful biomarker for prognostic stratification of Indian COVID-19 patients who progressed to acute respiratory distress syndrome (ARDS). Systemic hyperinflammation is a feature of severe COVID-19 disease and is often associated with ARDS (67). Their study showed that patients with severe COVID-19 ARDS had higher plasma suPAR abundance and that suPAR levels are associated with characteristic plasma proteomes, coagulation disorders and complement activation. Soluble uPAR may be the key link between the abnormally enlarged circulating myeloid cell compartment in critically ill COVID-19 patients and the systemic hyperinflammatory and hypercoagulable states encountered in these patients. However, influencing factors, including ethnic differences, remain to be further validated. A recent study identified an intriguing characterization of the pathophysiology of SARS-CoV-2 infection through multiplex and ultrasensitive proteomic analysis with inflammatory and cardiometabolic processes (63). They classified the plasma specimens of COVID-19 patients in the emergency department (ED) as undiagnosed suspected patients, confirmed but nonhospitalized patients, confirmed hospitalized patients, and patients with minor illnesses not related to SARS-CoV-2 and analyzed the correlation between 177 inflammation-related circulating proteins and cardiovascular disease using the proximity extension assay (PEA, Olink) technique. The results showed that 14 proteins were highly related to SARS-CoV-2 infection, and 12 proteins were further screened with highly significant levels (p < 0.001) that were closely related to hospitalization outcomes; among them, the proteins ADM, IL-6, MCP-3, TRAIL-R2, and PD-L1 can be used as predictors of death. Based on these markers, they constructed a composite risk fingerprint that effectively predicted mortality in their cohort of COVID-19 patients. Notably, they highlighted several hitherto unrecognized proteins: including the immunosuppressive receptor ligand PD-L1 which was specific to COVID-19, and the circulating hormone ADM which was recognized as the strongest single predictive markers in lethal progression. These findings provide the basis for informed risk stratification and alternative intervention, and give us a broader perspective at the level of monitoring inflammatory mediators.

**Table 1:** Potential body fluid Protein Biomarkers of COVID-19.

Matrix	Platform	Biomarker	Potential	Reference
Plasma	TMT 11-plex and LC-MS/MS, ELISA	ORM1, ORM2, S100A9, SERPINA3, PI16, LCP1, FETUB, AZGP1, CRP, CFI, CETP	Distinguished and predicted COVID-19 results	37
Serum	TMTpro (16plex) and UPLC-MS/MS	SAA2, ALB, CRP, SAA1, HABP2, HP, SERPINA10, CPN1, F5, SERPING1, SERPINA3, LUM, LRG1, ITIH3, CLEC3B, LBP, PGLYRP2, CFB, C6, LCP1, OAF, C9	Disease severity evaluation	38
Serum and plasma	A redesigned high-throughput MS platform	A1BG, ACTB, ACTG1, ALB, APOA1, APOC1, C1R, C1S, C8A, CD14, CFB, CFH, CFI, CRP, FGA, FGB, FGG, GSN, HP, ITIH3, ITIH4, LBP, LGALS3BP, LRG1, SAA1, SAA1, SAA2, SERPINA10, TF	Disease severity evaluation	60
Serum	SWATH-MS	SELENOP and PON1, CBP2, PZP	predictive value for disease recovery, severity and symptomatology	61
Plasma	LC-MS/MS SWATH	suPAR	Clinical outcomes stratification in severe COVID-19 patients with ARDS	62
Plasma	PEA, Olink	ADM, IL-6, MCP-3, TRAIL-R2, PD-L1	Prediction of COVID-19 Death Outcomes	63
Urine	TIMS-TOF MS with PASEF	MT1G, LPL, β2M, PRKACA, FOLR2, APOA4, IGLV3-25, EEF1A1	potential diagnostic markers	64
Urine and Serum	PRM-based targeted MS	IGF1, SAA1, FBLN5, APMAP, F9, SERPINA3, VWF, LEAP2, IGFBP3, IL1R2, TKT, VSIG4, IL6R, ALB, IGFBP2, RNASE4, MRC1, AGT, CR2, SHH	classifying and predicting COVID-19 severity	65

Urine is a more accessible source of body fluids and serves as a noninvasive biological sample from the peripheral circulation. Urinary proteomics has previously been reported in COVID-19 infection studies (64, 68). A recent study found that more detectable proteins and cytokines could be obtained in urine samples than in serum samples. Although some proteins that overlapped with serum showed opposite expression patterns, urine was comparable to serum in terms of the classification of COVID-19 as mild and severe (65). The study found that the levels of two important regulators involved in renal tubular reabsorption, megalin (LRP2) and cubilin (CUBN), showed a decreasing trend in the urine of patients with COVID-19. The researchers speculated that the renal tubular reabsorption process in COVID-19 patients may be disturbed, resulting in certain protein changes in urine and showing different expression patterns from those in blood. This phenomenon may also exist in other diseases, and further research is needed. In urine, CXCL14 was significantly correlated with lymphocyte counts in COVID-19 patients, which may be used to indicate the severity of COVID-19. Strikingly, the study also found that some proteins related to viral budding in the urine proteome showed significantly decreased levels in the urine of COVID-19 patients and were not detected in the serum. The above results indicate that the urine proteome showed higher detection sensitivity than the blood proteome in this study, and the urine proteomic analysis described in this study provides a novel and convincing platform for evaluating the immunopathobiology and clinical course of COVID-19.

## Conclusion and perspectives

Numerous studies have searched for proteomic biomarkers that are associated with the diagnostics and prognostics of COVID-19. Because mass spectrometry (MS)-based proteomics has been recognized as an ideal technique to detect infection-related changes in proteins (59) the MS technique has been widely used to explore various aspects of viral and host infection during the SARS-CoV-2 pandemic to date. A focus of proteomic research is protein−protein interactions (PPIs), and multiple studies have revealed extensive SARS-CoV-2 virus−host protein interaction networks through MS techniques, such as affinity purification mass spectrometry (AP-MS) and the complementary proximity-based labeling MS method (BioID-MS) (25, 69–77). Taken together, these studies have broadened our knowledge and understanding of the physiopathological profiles of SARS-CoV-2 infection and have provided large public data resources for reference.

At present, nucleic acid detection technology remains the mainstream method and gold standard for the diagnosis of SARS-CoV-2 infection. However, protein-based detection methods have been attempted, such as the SARS-CoV-2 antigen self-test kit, which is gradually becoming popular in some countries and regions. The SARS-CoV-2 antigen self-test kit principally consists of a rapid test for N protein antigen on the surface of virus particles (78). Compared with nucleic acid testing, antigen testing is a rapid and convenient test that can save labor costs associated with medical personnel and thus reduce the pressure on the health care system, especially during large-scale outbreaks. For the continuous emergence of new variants, the protective efficacy of existing vaccines is limited; thus, the design of new vaccines and preventive and control measures remain a hot issue for research. Several neutralizing antibodies and oral antivirals have been introduced for the treatment of COVID-19, and several other highly anticipated therapeutic agents have been reported to be in mid-stage clinical trials. Possible therapeutic agents and drug targets with potential for SARS-CoV-2 infection are still being explored (79–81). Proteomics and technology, as important weapons, will continue to play a great role in challenging disease and research fields.

The worldwide infection of SARS-CoV-2 and the various complications and sequelae caused by COVID-19 are still huge challenges faced by the world. As a recent article published in The Lancet pointed out, ‘the epidemic may end but COVID-19 will not’ (82). A number of COVID-19 proteomic markers have been identified since late 2019, and the combination of viral particle-based mass spectrometry studies, cell- and tissue-based studies of infection mechanisms, fluid- and disease-course-based studies, and studies of organismal immunity and cytokines have brought a clearer perspective. However, our understanding of the virus is still limited; with the emergence of OMICRON variants, there will be more mysteries to be explored further. Additionally, as the pandemic enters its third year, there is still limited understanding of the long-term health effects and sequelae of COVID-19 in patients who have recovered from the infection. Since proteomics can provide detailed information on SARS-CoV-2 structure and function with a comprehensive protein interaction network, and mass spectrometry-based proteomics has the advantage of high throughput without bias, proteomics will remain a powerful tool for fighting COVID-19 in the future.

## Author contributions

SY and JS conceived the idea and drafted the original manuscript. ZX and SY prepared the figure and table. XL and LS provided critical feedback. All authors contributed to the article and approved the submitted version.

## Funding

This work was supported by the Science and Technology Research Project of the Education Department of Jilin Province (JJKH20211181KJ/JJKH20201027KJ/JJKH20211149KJ).

## Conflict of interest

The authors declare that the research was conducted in the absence of any commercial or financial relationships that could be construed as a potential conflict of interest.

## Publisher’s note

All claims expressed in this article are solely those of the authors and do not necessarily represent those of their affiliated organizations, or those of the publisher, the editors and the reviewers. Any product that may be evaluated in this article, or claim that may be made by its manufacturer, is not guaranteed or endorsed by the publisher.
